# Genetic sources and loci for Fusarium head blight resistance in bread wheat

**DOI:** 10.3389/fgene.2022.988264

**Published:** 2022-09-30

**Authors:** Lei Wu, Xinyao He, Yi He, Peng Jiang, Kaijie Xu, Xu Zhang, Pawan K. Singh

**Affiliations:** ^1^ CIMMYT-JAAS Joint Center for Wheat Diseases, Jiangsu Academy of Agricultural Sciences, Nanjing, China; ^2^ International Maize and Wheat Improvement Center (CIMMYT), Texcoco, Mexico; ^3^ Institute of Cotton Research, Chinese Academy of Agricultural Sciences, Anyang, China

**Keywords:** Fusarium head blight, resistance, genetic sources, genome-wide association study, deoxynivalenol

## Abstract

Fusarium head blight (FHB) of wheat is an important disease worldwide, affecting the yield, end-use quality and threatening food safety. Genetic resources or stable loci for FHB resistance are still limited in breeding programs. A panel of 265 bread wheat accessions from China, CIMMYT-Mexico and other countries was screened for FHB resistance under 5 field experiments in Mexico and China, and a genome-wide association analysis was performed to identify QTLs associated with FHB resistance. The major locus *Fhb1* was significantly associated with FHB severity and Deoxynivalenol content in grains. FHB screening experiments in multiple environments showed that *Fhb1*-harbouring accessions Sumai3, Sumai5, Ningmai9, Yangmai18 and Tokai66 had low FHB index, disease severity and DON content in grains in response to different *Fusarium* species and ecological conditions in Mexico and China. Accessions Klein Don Enrique, Chuko and Yumai34 did not have *Fhb1* but still showed good FHB resistance and low mycotoxin accumulation. Sixteen loci associated with FHB resistance or DON content in grains were identified on chromosomes 1A, 1B, 2B, 3A, 3D, 4B, 4D, 5A, 5B, 7A, and 7B in multiple environments, explaining phenotypic variation of 4.43–10.49%. The sources with good FHB resistance reported here could be used in breeding programs for resistance improvement in Mexico and China, and the significant loci could be further studied and introgressed for resistance improvement against FHB and mycotoxin accumulation in grains.

## Introduction

Fusarium head blight (FHB) is one of the most devastating diseases of wheat and other small grain cereals, threatening global wheat production and food security and safety (Bai and Shaner, 2004; Ma et al., 2020). Many *Fusarium* spp. can infect wheat head, of which *F. graminearum* species complex (FGSC) is the predominant FHB pathogen in most wheat production areas (Van Der Lee et al., 2015). The distribution of various FGSC sub-species depends on geographies, climatic conditions and cropping systems, e.g., *F. asiaticum* is mainly in East Asia, while *F. meridionale* and *F. boothii* are mainly in South America and Africa (Zhang et al., 2012; Backhouse, 2014; Van Der Lee et al., 2015). FHB leads to severe yield loss, poor grain quality, and more importantly contamination of the infected grains with mycotoxins like deoxynivalenol (DON) or nivalenol, for which public concerns have prompted governments to set upper limits for DON in wheat grain and its products (Mesterházy et al., 1999; He et al., 2016; Ma et al., 2020).

Breeding for FHB resistance by using QTL/genes in genetic sources is one of the effective approaches to control this disease and prevent toxins contamination in grains after harvest. FHB resistance in wheat is a complex quantitative trait with strong genotype-by-environment interactions, resulting in different response to *Fusarium* pathogens across different environments (Mesterhazy, 2020). Host resistance to FHB involves five resistant types, i.e. Type I for initial infection, Type II for disease spread, Type III for toxin accumulation, Type IV for kernel infection, and Type V for yield reduction (Schroeder and Christensen, 1963; Miller and Amison, 1986; Mesterhazy, 1995). Type II and III resistance have been widely studied (Buerstmayr et al., 2020; Ma et al., 2020; Mesterhazy, 2020), and over 600 loci for Type II or III resistance have been mapped on all 21 wheat chromosomes (Venske et al., 2019; Zheng et al., 2021). Of the nominated *Fhb* genes, *Fhb1* (Cuthbert et al., 2006; Liu et al., 2006), *Fhb2* (Cuthbert et al., 2007), *Fhb4* (Xue et al., 2010) and *Fhb5* (syn. *Qfhs.ifa-5A*) (Xue et al., 2011; Buerstmayr et al., 2018; Steiner et al., 2019) are derived from common wheat, of which the former two mainly confer Type II resistance and the latter two mainly confer Type I resistance. The wild relatives of wheat have also contributed several resistance genes/loci, like *Fhb3* (Qi et al., 2008) from *Leymus racemosus*, *Fhb6* (Cainong et al., 2015) from *Elymus tsukushiensis* and *Fhb7* (Guo et al., 2015) from *Thinopyrum elongatum*, all conferring Type II resistance. Type II and Type III resistance are usually highly correlated in field experiments as observed by most researchers, although QTL exclusively for Type III resistance have been reported (He et al., 2019).

Thousands of wheat accessions have been screened for FHB resistance throughout the world over the last decades, but resistant sources for FHB improvement in breeding programs remain limited. *Fhb1* (Syn. *Qfhs.ndsu-3BS*) derived from Sumai3 or its derivative Ning7840 is recognized as a locus with major effect and stable resistance and has been widely used in wheat breeding programs worldwide (Zhu et al., 2019; Buerstmayr et al., 2020; Hao et al., 2020; Ma et al., 2020; Gaire et al., 2022b). Several research groups have cloned the candidate genes of *Fhb1* (Rawat et al., 2016; Li et al., 2019; Su et al., 2019) and designed diagnostic markers for marker-assisted selection (MAS) (He et al., 2018; Su et al., 2018; Singh et al., 2019). Sumai3 that has *Fhb1*, *Fhb2*, and *Fhb5* is a widely used donor for FHB resistance improvement, and more than 20 released spring wheat varieties have Sumai3 in their pedigrees in North America and Canada (Zhu et al., 2019; Ghimire et al., 2020). The donor parent of *Fhb1* in the Chinese wheat breeding programs is, however, not Sumai3 but Ningmai9 (developed by Jiangsu Academy of Agriculture Sciences, JAAS) because of its better agronomic traits and high yield potential (Zhu et al., 2018). More recently, *Fhb7* derived from *Th. elongatum* was reported to confer broad resistance to *Fusarium* species by detoxifying DON without yield penalty (Wang et al., 2020), and several locally adaptive lines with *Fhb7* in their pedigrees have been tested in Regional Yield Trials in China (Prof. Hongwei Wang, Shandong Agricultural University, personal communication).

Despite the achievement, highly resistant sources and major loci for FHB resistance are scarce for wheat breeding, it is therefore worthwhile to identify additional FHB resistance sources and loci for gene pyramiding. The objectives of this study were to screen a collection of common wheat varieties and elite breeding lines of worldwide origin for FHB resistance in Mexico and China, and to conduct a genome-wide association study (GWAS) to identify QTL for FHB resistance in field experiments.

## Materials and methods

### Plant materials

In this study, 265 wheat accessions (CIMMYT-China panel) were screened for FHB resistance under field conditions in Mexico and China. The panel included commercial varieties and breeding lines of worldwide origin, i.e., 131 Chinese accessions mainly from the Yellow and Huai River Valley Region and Middle-lower Yangtze Valley Region, 71 from CIMMYT-Mexico, 41 from South America, 10 from North America, five each from Asia and Europe, and one each from Oceania and Africa (Supplementary Table S1). The accessions were mostly of spring type along with a few of winter type. In Mexico, Sumai3 and Heilo were used as resistant checks, while Gamenya and Ocoroni were included as susceptible checks. In China, Sumai3 and Yangmai158 served as resistant checks with Annong8455 and Jimai22 as susceptible checks.

### Field trials and Fusarium head blight screening

The CIMMYT-China panel was evaluated for FHB resistance in CIMMYT’s El Batan research station (altitude of 2,240 masl, 19.5^º^N/98.8^º^W, with an average annual precipitation of 625 mm) in Mexico, and JAAS’s FHB nursery (altitude of 22 masl, 32.0^º^N/118.8^º^E, with an annual precipitation of 800–1034 mm) in Nanjing, China. In each location, the accessions were planted in 1-m double rows with two replications in randomized complete block design. The trials were conducted in the 2018 and 2019 cropping cycles (from May to September) in Mexico, and in the 2018–19, 2019–20, and 2020–21 cropping cycles (i.e. November to May) in China.

In Mexico, inoculum comprised a mixture of five *F. graminearum* isolates, CIMFU No. 85, 89, 108, 162, and 222, following the protocols described by (He et al., 2013). These isolates were obtained from naturally infected wheat heads in the El Batan station in 2017 and were selected based on their high DON productivity and high aggressiveness in greenhouse experiments. At anthesis, the field plots were sprayed with an inoculum of 50,000 spores/ml, and the procedure was repeated 2 days later to reinforce the infection. A misting system was set up in the nursery, operational from 9 am to 8 pm with 10 min of spraying each hour, to maintain a humid environment conducive for FHB infection. Field evaluation of FHB infection was carried out at 25 days post-inoculation (dpi) on the 10 spikes tagged at anthesis (Feekes 10.5.1). Numbers of total and infected spikelets of each spike were scored for the calculation of FHB index with the formula: FHB index = severity × incidence, where severity means the averaged percentage of diseased spikelets, and incidence the percentage of symptomatic spikes. About 20g grain sample was ground with a coffee mill and a 2g sub-sample was measured for DON quantification with the Ridascreen Fast DON ELISA kit (RBiopharm GmbH, Darmstadt, Germany) following the manufacturer’s instructions.

In China, *F. asiaticum* isolates Fa0609, Fa1312 and Fa0980 were used in this study. These isolates were isolated from different wheat plots in JAAS (Nanjing, China) in 2006, 2009 and 2013, respectively, and had shown strong virulence in greenhouse and field experiments. The fungal isolates were applied to wheat kernels to produce spawn inoculum, which was distributed on the soil surface with a density of 30 g/m^2^ on the 20th day before flowering and the inoculation was repeated 2 weeks later. All the plots were misted for 15 min per hour during the day to create a humid environment for fungal development and spread. The numbers of diseased spikelets of 10 random spikes were recorded to calculate the disease severity for each accession at the milky ripe stage (Feekes 11.2). The spawn inoculation experiments were performed only during 2018–19 and 2019–20 cropping cycles. Besides, point inoculation with a single isolate Fa0609 was also conducted to evaluate the Type II resistance of the panel during 2018–19, 2019–20, and 2020–21 cropping cycles. Ten spikes per accession were inoculated via injecting 10 μl suspension of *F. asiaticum* (100,000 spores/ml) into a central spikelet at anthesis (Feekes 10.5.1), and then the spikes were covered with a plastic bag for 3 days to meet the moisture requirement for fungal infection. The moisturizing measure was the same as that mentioned above. The numbers of diseased spikelets were recorded as FHB severity on the 21st day after inoculation. The inoculated spikes were harvested to detect DON content with DON ELISA test kit (Huaan Magnech Bio-tech, China) using an indirect competitive enzyme-labeled immunoassay.

The phenotypic dataset including the mean values of FHB index, FHB severity and DON content in grains in each environment for each accession were used in subsequent analysis. The datasets of Mexico and China were calculated separately. Pearson’s correlation coefficients among environments were computed by R to test the correlation of FHB responses across different environments (R Team, 2011). Turkey’s mean comparison tests were performed among subgroups using R (R Team, 2011).

### Genotyping

The GWAS panel was genotyped with the DArTSeq technology at the Genetic Analysis Service for Agriculture (SAGA) at CIMMYT, Mexico. Markers with more than 10% missing data or minor allele frequency less than 1% were eliminated, resulting in 18,436 high quality markers, of which 14,195 SNPs with known physical positions in Chinese Spring reference genome v.1.0 were used for population structure and LD analysis. PCR-based marker JAASM395 was used to test whether an accession carried *Fhb1* (Chinese patent, ZL201811515195.8), and the primer pair was as follows: JAASM395F: GTC​TCC​GTT​CAA​TTC​GGT​GAG​T; JAASM395R: GAC​AAT​GTG​AAG​GCG​TTG​TCT​A. Analysis on population structure and linkage disequilibrium followed the method in (Wu et al., 2021). Briefly, a Bayesian model-based method was used to infer the number of subpopulation among all accessions with the software STRUCTURE v 2.3.4 (Pritchard et al., 2000). Five independent analyses for the assumed number of subpopulations (K value) from 1 to 8 were performed based on an admixture model with MCMC replications and burn-in time number set at 1×10^5^ and 1×10^4^, respectively (Pritchard et al., 2000). Optimal value of population size was inferred by CLUMPP based on the data of STRUCTURE (Jakobsson and Rosenberg, 2007). Pairwise LD (*r* ^2^) for all pairwise comparisons in a distance of 10,000 kb was estimated by the software PLINK (Chang et al., 2015) and were plotted against the physical distances, and then a nonlinear regression was fitted in R (R Team, 2011). The critical *r* ^2^ value was determined as the 95th quantile for all *r* ^2^ values between unlinked SNPs. The intersection between the critical *r* ^2^ value and the regression line was used to estimate the average size for LD blocks in this panel.

### Genome-wide association analysis

Genome-wide association for FHB resistance across environments was performed using software TASSEL v5.0 pipeline command line interface (Bradbury et al., 2007). Mixed linear models (MLM) with K/Q and K/P matrices as covariates were chosen for all GWAS analyses. The Q matrix was calculated and estimated by STRUCTURE v2.3.4 (Pritchard et al., 2000). Physical positions of significant SNPs were determined by sequence alignments with “Chinese Spring” reference genome v1.0 on the website of EnsemblPlants (http://plants.ensembl.org/index.html) using BLAST program with default parameters. Physical positions of significant MTAs were compared to meta-QTL reported by (Zheng et al., 2021) to see their novelty.

## Results

### Fusarium head blight screening in Mexico and China

**FIGURE 1:**
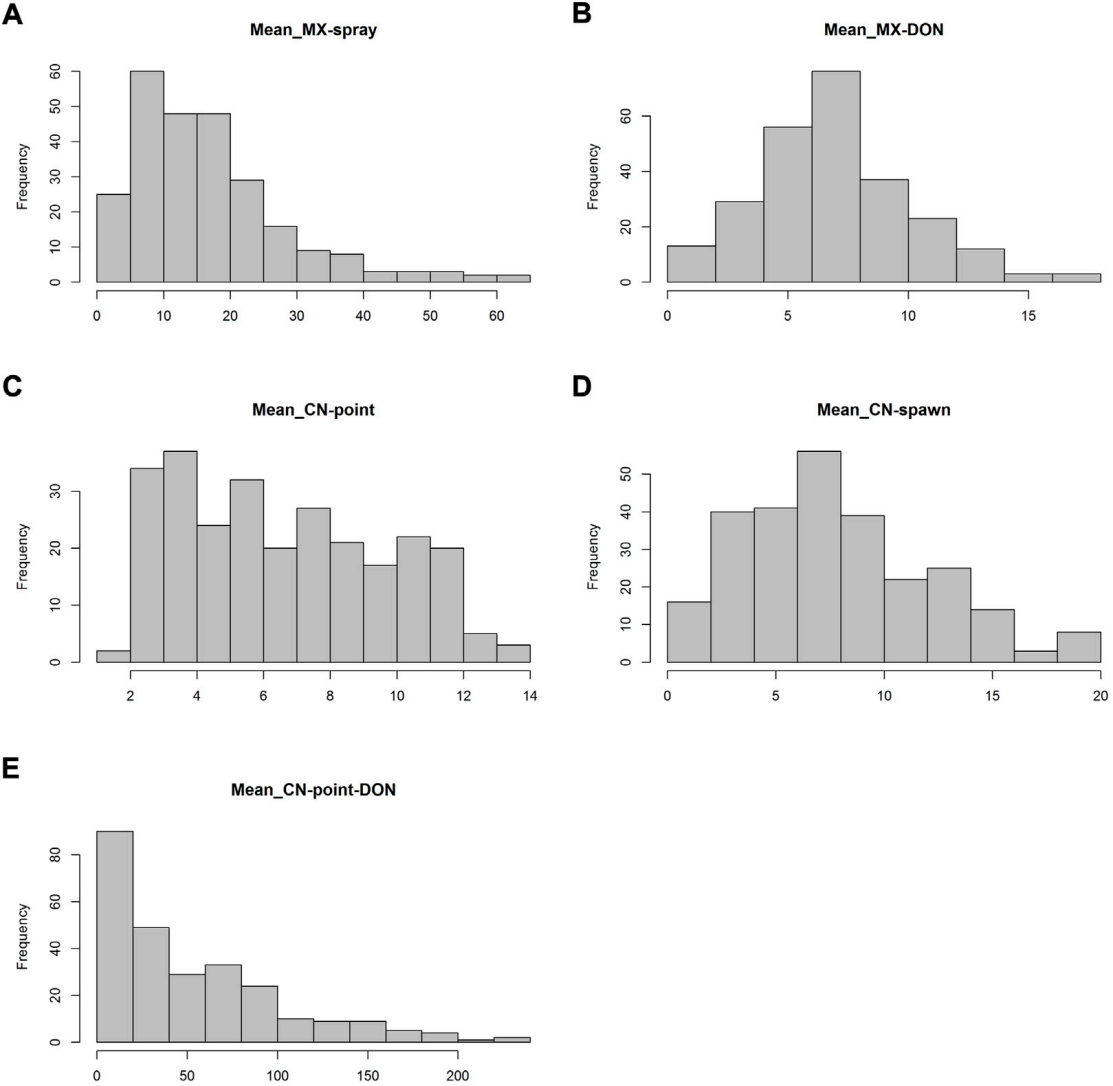
Frequency distribution of FHB index **(A)**, number of diseased spikelets **(C and D)** and DON content in grains **(B and E)** based on the mean data in Mexico (MX, spray inoculation) and China (CN, point and spawn inoculation).

In spray inoculation experiments in Mexico, FHB index showed a skewed distribution toward the low disease direction and the panel exhibited a grand mean FHB index of 16.83%. The accession Ning894013 had the lowest FHB index of 0.97%, while Norseman had the maximum of FHB index of 62.87% (Figure 1A, Supplementary Table S1). DON content in grains showed a continuous distribution with a mean of 6.91 μg/g (Figure 1B, Supplementary Table S1). In point inoculation experiments in China, numbers of diseased spikelets evenly distributed ranging from 2 to 13 with a mean of 6.6 (Figure 1C, Supplementary Table S1). In spawn inoculation experiments, the mean numbers of diseased spikelets were higher than that in point inoculation experiments (Figure 1D). The DON content in grains in point inoculation experiments was skewed to the low content direction, though, the mean value 54.11 μg/g was much higher than that in spray inoculation experiments (Figure 1E, Supplementary Table S1).

Phenotypic correlation varied greatly among different experiments, from low non-significant correlation (r = 0.07) to high significant correlation (r = 0.79). Generally, low to moderate correlations were observed among different inoculation methods, and the highest correlation was found between number of diseased spikelets and DON content in point inoculation experiments in China (Table 1).

**TABLE 1:** Pearson’s correlation coefficients among experiments in Mexico (MX) and China (CN).

	MX.spray	MX.DON	CN.point	CN.spawn
MX.DON	0.32*	0.44*	0.34*	0.33*
CN.point	0.09			
CN.spawn	0.44*	0.47*		
CN.pointDON	0.07	0.45*	0.79*	

Mean values across years were used here for FHB, index (spray inoculation in Mexico), number of diseased spikelets (point and spawn inoculation in China), DON, content in Mexico and China. * indicates significant correlations at *p* < 0.001.

Genetic resources with moderate resistance to FHB and low DON content in grains were listed in Table 2. As expected, *Fhb1*-carriers Sumai3, Sumai5, Ningmai9, Yangmai18 and Tokai66 exhibited good resistance in all experiments in Mexico and China. Accessions Klein Don Enrique, Chuko and Yumai34 that do not have *Fhb1* still showed good FHB resistance and low toxin accumulation.

**TABLE 2:** Top performers in FHB and DON traits across experiments.

Name	Origin	Group	*Fhb1*	FHB index (%)	Number of disease spikelets		DON content (μg/g)	
				Spray inoculation	Point inoculation	Spawn inoculation	Point inoculation	Spray inoculation
Klein Don Enrique	Argentina	2A	No	3.34	3.33	1.60	9.79	2.75
Chuko	Japan	2B	No	5.72	3.88	2.13	6.94	2.45
Tokai66	Japan	2B	Yes	5.79	3.46	2.22	6.12	1.05
Sumai3	China	2C	Yes	7.21	3.48	1.13	11.33	1.24
7P3	China	2C	Yes	1.14	2.85	2.29	4.99	0.74
Ningmai18	China	2B	Yes	7.91	3.80	3.50	14.42	3.68
Ningmai9	China	2C	Yes	5.25	2.30	2.39	6.07	3.80
Ningyan1	China	2C	Yes	1.89	2.10	1.58	3.55	7.48a
Sumai5	China	2C	Yes	7.57	2.90	3.50	24.18a	5.35a
Yangmai18	China	2B	Yes	5.73	2.25	7.24a	8.82	3.68
Yumai34	China	2C	No	6.88	2.65	2.50	26.42a	4.60

Numbers marked with “a” rank between the first and second quantiles in their own datasets.

Population structure analysis indicated that the panel could be divided into two subpopulations, which could be further divided into five groups 1A, 1B, 2A, 2B and 2C. The clustering of subpopulations mainly associated with geographical origins. Subpopulation 1 (130 accessions) involved accessions mainly from CIMMYT, South America, North America, and Europe, while most accessions in subpopulation 2 (135 accessions) were from Asian countries including China, Japan and India (Supplementary Table S1).

**FIGURE 2:**
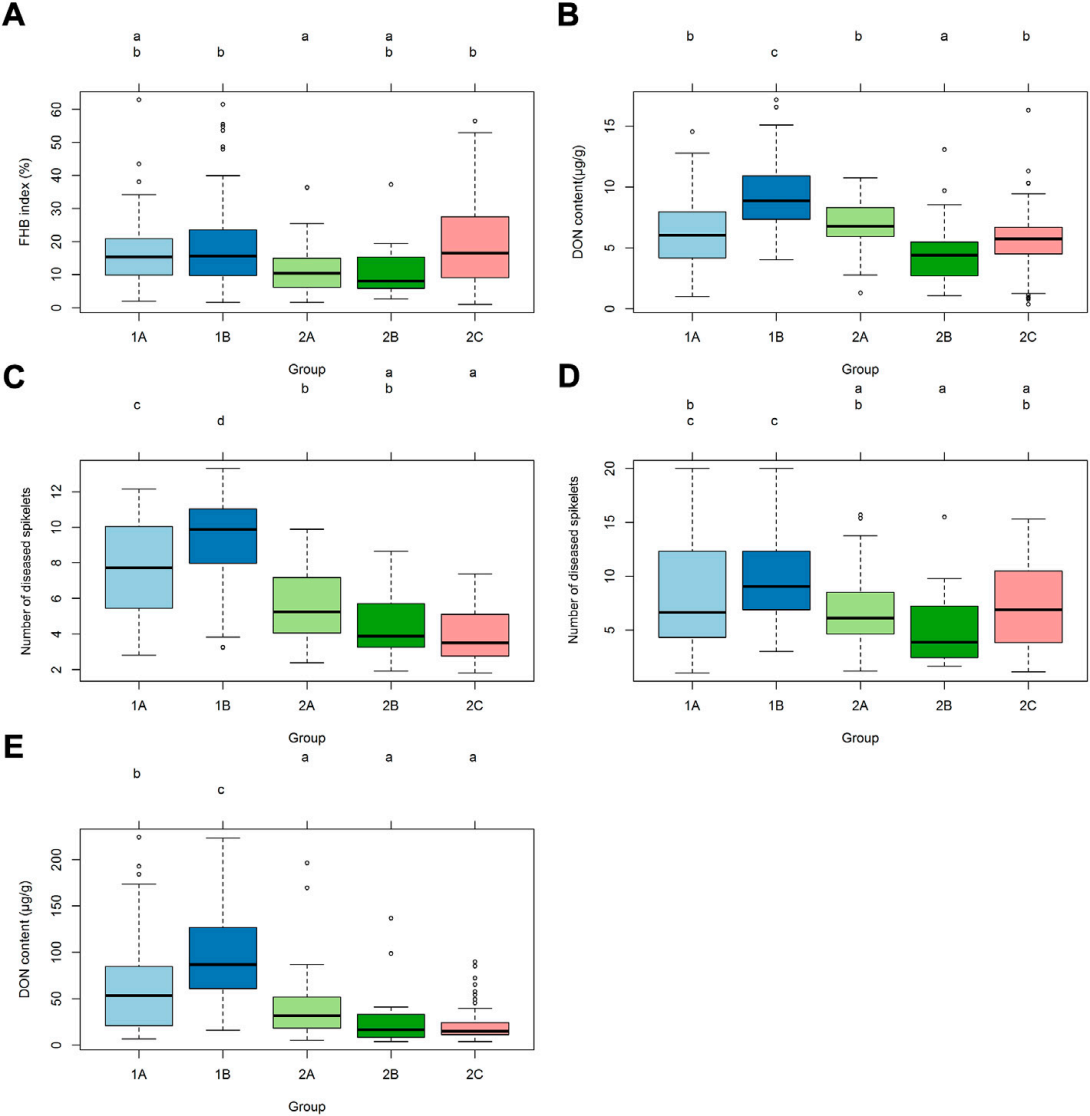
Distributions of FHB index, number of diseased spikelets and DON content among different groups. **(A)** Distribution of mean data of FHB index using spray inoculation among different groups in 2018 and 2019 in Mexico. **(B)** Distribution of mean data of DON content using spray inoculation among different groups in 2018 and 2019 in Mexico. **(C)** Distribution of mean data of FHB severity using point inoculation among different groups in 2018, 2019 and 2020 in China. **(D)** Distribution of mean data of FHB severity using spawn inoculation among different groups in 2018 and 2019 in China. **(E)** Distribution of mean data of DON content in grains using point inoculation among different groups in 2018, 2019 and 2020 in China.

There were significant differences in FHB resistance or DON content in grains among the groups 1A, 1B, 2A, 2B and 2C (*p* < 0.01) (Figures 2A–E). In spray inoculation experiments in Mexico, group 2A had lower mean FHB index than 1B and 2C, and 2B had the lowest DON content in grains (Figures 2A,B). In the experiments in China, however, subpopulation 2 outperformed subpopulation 1 significantly in most cases, and significant differences were also found within subpopulations (Figures 2C–E).

**FIGURE 3:**
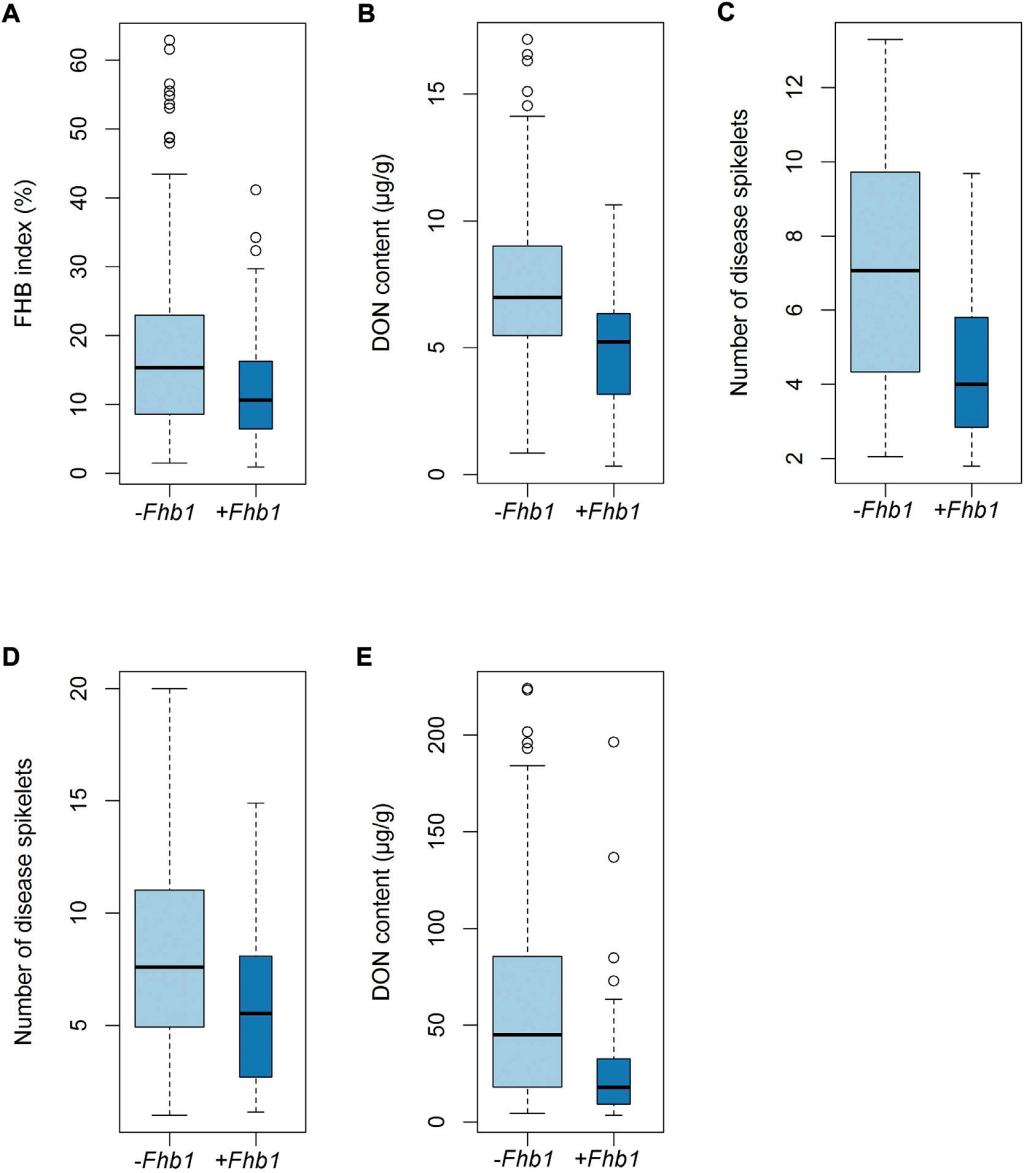
Differences in FHB index (A), number of diseased spikelets (C and D) and DON content (B and E) between *Fhb1* and non-*Fhb1* accessions.

Significant differences in FHB index or number of diseased spikelets were observed between *Fhb1* and non-*Fhb1* groups, where the former outperformed the latter; the same trend applied to DON content in both Mexico and China (Figure 3).

### Loci associated with Fusarium head blight resistance

**FIGURE 4:**
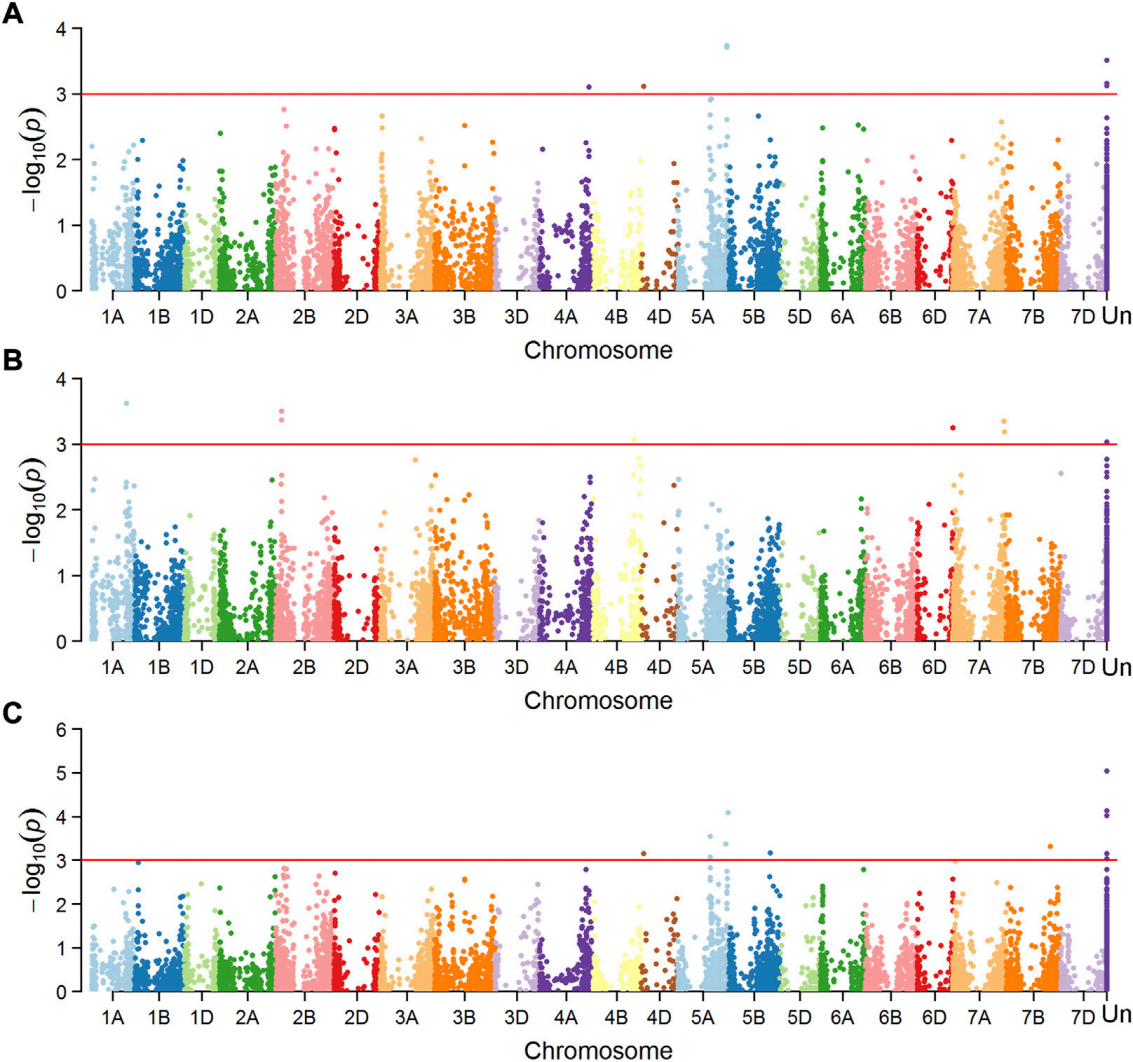
Manhattan plots showing SNPs associated with number of diseased spikelets using point inoculation **(A)** and spawn inoculation **(B)**, and those for DON content using point inoculation **(C)** in China.

**FIGURE 5:**
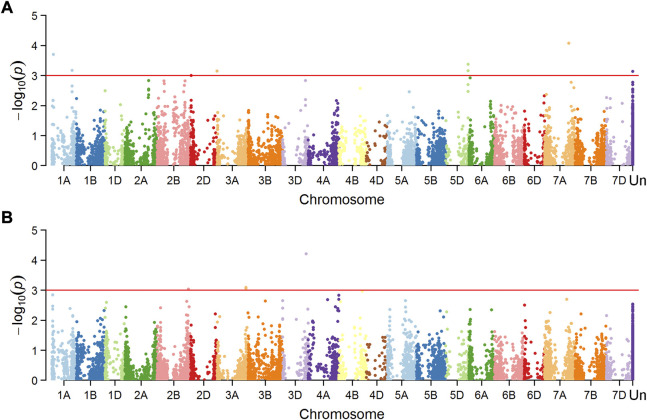
Manhattan plots showing SNPs associated with FHB index using spray inoculation **(A)** and those for DON content **(B)** using spray inoculation in Mexico.

Marker-trait associations (MTAs) were tested separately for the dataset of mean value in Mexico and China, and a total of 16 MTAs (24 SNPs) were significantly associated with FHB resistance or DON content in grains (Figures 4, 5), explaining phenotypic variation between 4.43% and 10.49% (Table 3). Four of the SNPs located in annotated gene regions without known functions. Ten MTAs (18 SNPs) on chromosomes 1A, 1B, 2B, 7A, 4D, 4B, 5A and 7B were significantly associated with FHB resistance (*p* < 0.001), of which markers 3026949, 979146, 1157139 and 10334520 were significantly associated with both FHB resistance and DON content using point inoculation data in China. Marker 1044062 on 4B chromosome was identified in association with FHB resistance in China and DON content in Mexico. Only one marker 1099971 was significantly associated with FHB resistance in both China and Mexico, overlapping a reported mQTL sMQTL-1A-5. Six markers on chromosomes 2B, 3A, 3D, 4D, 5A and 5B were associated with only DON content across China and Mexico (Table 3). Significant markers for FHB resistance or DON content in individual environments were listed in Supplementary Table S2, explaining phenotypic variation of 4.34–11.33%.

**TABLE 3:** Markers significantly (-log10(*p*) ≥3.0) associated with FHB resistance and DON content.

Marker id	SNP	Chr	Position (bp)	R^2^ (%)	RAF (%)	Overlapping gene	Overlapping mQTL	Model	Environments
FHB resistance									
4910975	A/G	1A	31918043	7.26	15.66	NA	sMQTL-1A-3	P+K; Q+K	MX-spray
1099971a	C/T	1A	480934139	5.40	21.26	TraesCS1A02G283400	sMQTL-1A-5	P+K; Q+K	CN-spawn; MX-spray
1081753	T/C	1B	258837217	7.04	5.63	NA	NA	P+K; Q+K	MX-spray
1055088	T/G	2B	73002236	5.76	48.58	NA	NA	P+K; Q+K	CN-spawn
1044062	A/G	4B	572558191	4.69	8.12	NA	NA	P+K; Q+K	CN-spawn; MX-DON
3026949	A/G	4D	35405745	4.43	4.31	NA	NA	P+K	CN-point; CN-pointDON
979146	G/A	5A	466024980	5.56	94.62	NA	sMQTL-5A-5	Q+K	CN-point; CN-pointDON
1157139b	T/C	5A	697068377	8.27	6.05	NA	sMQTL-5A-8	P+K; Q+K	CN-point; CN-pointDON
1229379	G/C	7A	706764943	5.73	18.11	TraesCS7A02G524200	NA	P+K; Q+K	CN-spawn
1034520	C/A	7B	701319079	10.49	19.60	NA	NA	P+K; Q+K	CN-point; CN-pointDON
DON content									
3956613	G/A	2B	753628598	7.00	21.20	TraesCS2B02G559400	NA	P+K; Q+K	MX-DON
1285715	T/C	3A	705290933	6.01	67.57	NA	NA	P+K; Q+K	MX-DON
1218288	A/G	3D	571054888	7.21	68.25	NA	NA	P+K; Q+K	MX-DON
1091396	T/C	4D	502708990	5.27	6.92	TraesCS4D02G350500	NA	P+K	CN-pointDON
989900	C/A	5A	666683794	4.73	90.77	NA	sMQTL-5A-7	Q+K	CN-pointDON
1091498	C/T	5B	571213517	6.92	65.32	NA	sMQTL-5B-4	Q+K	CN-pointDON; MX-DON

1099971a, significant markers 1099971 and 3064923 located on the same locus based on the LD, analysis. 1157139b, significant markers 1157139, 1217190, 5050428, 2259167, 5371234, 982983, 1100295 and 1091475 located on the same locus based on LD analysis. Physical positions for the associated SNPs were based on Chinese Spring reference genome v1.0. Physical positions of the significant SNPs were compared to metaQTL intervals reported in Zheng et al., 2021. RAF refers to the resistance allele frequencies of the associated SNPs, and R^2^ refers to the phenotypic variation explained by the associated SNPs.

## Discussion

FHB of wheat is a serious disease in the temperate and humid regions around the world. Screening for resistant sources is a prerequisite for the improvement of FHB resistance, but many factors affect the development of locally adapted resistant varieties, including complex inheritance, multiple resistance types, difficulties on precise phenotyping, association of FHB resistance with undesirable traits (late and tall plant phenology etc.), and strong genotype-by-environment interaction. In the present study, 265 accessions from China, CIMMYT-Mexico and other countries were screened for FHB resistance in Mexico and China. FHB index and disease severity were used to evaluate FHB resistance of all the accessions in field trials, and then DON content in infected grains was detected. Spray inoculation simulates the process of disease development under natural conditions in Mexico, and the FHB index showed a combination of Types I and II resistance to FHB. Therefore, the data of FHB incidence does not refer to strictly Type I resistance due to the late scoring time. FHB severity is characterized only for Type II resistance by the point inoculation method in China, which is considered a stable and accurate screening method. There was no significant correlation between the data of FHB index in Mexico and FHB severity in China (Table 1), which was caused by accessions with contrasting resistance components in the two countries, e.g., several Chinese accessions showed good Type II resistance in China but poor Type I resistance in Mexico (Supplementary Table S1). The reason could be ascribed to the relatively high frequency of *Fhb1* (34.1%) conferring good Type II resistance in Chinese accessions. Uncontrollable factors such as environmental change and inoculum content make accurate phenotyping of Type I resistance difficult. Type II resistance evaluation is mandatory before the release of Chinese varieties, while other resistance components including Type I resistance are optional in breeding program. However, accessions with good Type II resistance but poor Type I resistance could still suffer high yield loss and DON contamination under natural conditions with high *Fusarium* pressure. Pyramiding of Types I and II resistance to FHB will be a promising breeding strategy, and introgression of *Fhb1*, *Fhb4* and *Fhb5* into five modern Chinese wheat reduced FHB severity by 95% without penalty for agronomic traits and yield (Zhang et al., 2021). In Mexico, toxin content was moderately correlated with the FHB index, while in China, grain toxin levels were highly correlated with Type II resistance (Table 1). These data suggested that DON content in grains actually represented a combination of Type I/II and III resistance, which may explain why QTLs exclusively associated with Type III resistance are rarely mapped (He et al., 2019).

Most of the wheat varieties in the middle and lower reaches of the Yangtze river in China have *Fhb1* that confers moderate resistance to FHB (data not shown), otherwise it would not be released in National Wheat Production Trials. Recently, the development of wheat germplasm combining *Fhb1* and *Sr2* in CIMMYT backgrounds would be used in breeding for both FHB and stem rust resistance (He et al., 2020). Large quantities of QTL mapping and omics data for FHB resistance have been released in the previous reports, it is widely accepted that *Fhb1* on 3BS chromosome is a major and stable locus for Type II resistance to FHB (Cuthbert et al., 2006; Liu et al., 2006; Gunnaiah et al., 2012; Schweiger et al., 2013; Schweiger et al., 2016; Eldakak et al., 2018). Previous studies have shown that *Fhb1* did not effectively increase resistance to FHB in certain genotypes (Pumphrey et al., 2007), and a similar case has been observed in our data, that is, several accessions harboring *Fhb1* showed moderate susceptibility to FHB (Supplementary Table S1). The additive effect of minor loci might play an important role in FHB resistance, and non-*Fhb1* accessions with moderate susceptibility to resistance could still be used in breeding or genetic analysis (Supplementary Table S1). Totally 11 accessions exhibited stable moderate resistance and low DON content in grains (Table 2). It was worth noting that three accessions Klein Don Enrique, Chuko and Yumai34 did not contain *Fhb1*, but were resistant to multiple *Fusarium* species from Mexico and China. These non-*Fhb1* accessions with moderate resistance to FHB would facilitate their application in breeding and provide alternative options for FHB improvement.

The present study identified 16 genetic loci associated with FHB resistance and/or DON content in grains, 6 of which overlapped with reported metaQTL intervals (Zheng et al., 2021). Marker 4910975 is in the interval of previously reported smQTL-1A-3 (28–38 Mb) derived from “CJ9303” and two European winter wheats “History” and “Pirat” (Jiang et al., 2007; Holzapfel et al., 2008). Marker 1099971 is close to the flanking marker IWA7577 of smQTL-1A-5 (484–509 Mb) and overlapped with this QTL based on the LD data (Zheng et al., 2021). Marker 1099971 is found in the second exon of *TraesCS1A02G283400*, which is annotated as a copper ion-binding protein involved in lignin catalytic synthesis. Marker 979146 associated with Type II FHB resistance here overlaps with reported QTL interval of smQTL-5A-5 from “Wangshuibai” and CIMMYT wheat line “C615” (Ma et al., 2006; Yi et al., 2018). A cluster of SNPs tagged by marker 1157139 falls into the interval of smQTL-5A-8 (681–694 Mb), which is a minor QTL from Swiss winter wheat cultivar “Arina” (Paillard et al., 2004). A locus associated with DON content in grains tagged by marker 989900 is close to the flanking marker of smQTL-5A-7 (662-645 Mb) (Zheng et al., 2021), which has been associated with Types II and III resistance in different accessions by several independent research groups (Buerstmayr et al., 2011; Chu et al., 2011; Lu et al., 2013; Malihipour et al., 2017; Zhao et al., 2018). Similarly, marker 1091498 that was only associated with DON content in grains falls into a known QTL interval, too. Considering that these two loci were significant in point inoculation experiments, they are likely associated with Type II resistance, too (Zheng et al., 2021). The rest 10 loci are not tightly linked to the previously reported QTL through sequence alignments, implying that they may be novel for FHB resistance. Marker 1229379 associated with FHB resistance is located in the coding region of *TraesCS7A02G524200* on 7A, which could play a role in defense responses to pathogens by maintaining cell membrane integrity.

Our results, together with the large number of loci reported in previous studies (Arruda et al., 2016a; He et al., 2016; Buerstmayr et al., 2020; Ma et al., 2020; Zheng et al., 2021), suggest that multiple genes/loci are involved in FHB resistance under different environments, although their effects are mostly small and could not be easily used in MAS. Four MTAs (3026949, 979146, 1157139 and 1034520) were associated with both Type II resistance and DON content, which was expected due to the high correlation between grain toxin levels and Type II resistance, as described in the present study and previous reports (He et al., 2019; Zheng et al., 2021). Markers 1099971, 1044062 and 1091498 were identified in experiments in both China and Mexico, which may suggest their roles in response to different environmental factors and *Fusarium* species.

Most significant MTAs reported here appeared as single SNP markers, possibly due to the low resolution of genotyping. Similar cases have been reported by (Arruda et al., 2016a) and (Miedaner et al., 2011). Based on MTAs, genomic selection has shown intermediate to high prediction accuracy for FHB resistance traits, including DON content in grains, Fusarium damaged kernels and FHB severity (Arruda et al., 2016b; Gaire et al., 2022a). The effects of the markers reported here could be re-estimated in larger breeding populations and used in genomic selection for FHB resistance improvement.

## Conclusion

FHB screening experiments on 265 wheat accessions in Mexico and China showed that 11 accessions had stable FHB resistance or low DON content in grains, of which three accessions Klein Don Enrique, Chuko and Yumai34 did not contain *Fhb1*. Sixteen loci associated with FHB resistance or DON content in grains were identified on chromosomes 1A, 1B, 2B, 3A, 3D, 4B, 4D, 5A, 5B, 7A, and 7B in multiple environments, 6 of which overlapped with reported metaQTL. The genetic sources could be used in the breeding programs for FHB improvement, and the associated loci could be further mapped to identify better markers for MAS.

## Data Availability

The datasets presented in this study can be found in online repositories. The names of the repository/repositories and accession number(s) can be found in the article/Supplementary Material. Raw genotypic data is accessible via https://hdl.handle.net/11529/10548748.
